# “We Are One of the Most Depressed Professionals”: Nurses' Experiences of Psychological Hazards in A District Hospital

**DOI:** 10.1002/nop2.70597

**Published:** 2026-05-06

**Authors:** Perpetual N. B. Kodom, Feikoab Parimah, Collins Badu Agyemang

**Affiliations:** ^1^ Department of Social Sciences Central University Miotso Ghana; ^2^ Psychology Department University of Ghana Legon Ghana

**Keywords:** behaviour, deep acting, hazards, occupational, psychological, surface acting

## Abstract

**Aim:**

This study explored the occupational hazards faced by nurses in a district hospital in Ghana, analysed their psychological outcomes, and examined how emotional labour mediates nurses' behaviour in patient care.

**Design:**

A focused ethnographic study.

**Methods:**

Over the course of 11 months, data were collected purposively from 31 general nurses and one key informant using in‐depth interviews, participant observation, and informal dialogues. Data were analysed thematically using network analysis, supported by NVivo 12 Plus. Rigour was enhanced through prolonged engagement, triangulation, pilot testing, member checking, and peer debriefing.

**Results:**

Two categories of psychological hazards emerged: default hazards (unavoidable), such as patient deaths, ubiquity of sickness, and unpleasant sights; and escapable hazards (somewhat avoidable), including work‐related injuries and shift‐work maladaptation. These hazards triggered grief, disgust, fear, anxiety, and fatigue, which nurses managed through emotional labour strategies: surface acting, deep acting, spiritual coping, peer support, and emotional detachment. While these strategies enabled professionalism, they often led to emotional dissonance, burnout, and strained relationships between nurses and patients.

**Conclusion:**

Occupational hazards in hospital wards carry significant psychological burdens that shape nurses' behaviour and professional conduct. Emotional labour mediates these responses, but reliance on individual coping strategies is unsustainable without institutional support. Comprehensive support systems, such as psychotherapeutic counselling during and after nursing school, are needed to mitigate the effects of these occupational hazards on healthcare providers.

**Patient or Public Contribution:**

No patient or public contribution.

## 1 Introduction

The healthcare sector exposes workers to a wide range of occupational hazards, which are short‐ and long‐term risks associated with unhealthy workplace environments. These occur in biological, chemical, environmental, physical, and psychological forms (Kuş et al. 2024; Nankongnab et al. 2020). Among these, psychological hazards are particularly significant for nurses, as they stem from stressors such as work overload, bullying, poor interpersonal relationships, organisational injustice, and workplace violence (Kodom 2026; Okeafor and Alamina 2018). These hazards often manifest in stress, exhaustion, burnout, and job dissatisfaction, undermining nurses' well‐being and professional conduct (Che Huei et al. 2020; Soto‐Rubio et al. 2020).

To understand how nurses navigate such hazards, this study is guided by Hochschild's (1983) theory of emotional labour, which explains how workers regulate their emotions to meet professional expectations. Emotional labour involves adhering to feeling rules and employing strategies such as surface acting, which involves suppressing or simulating emotions to appear professional, and deep acting, which involves genuinely inducing emotions to sustain care (Hochschild 2003). This framework highlights the psychological costs of emotional regulation, including emotional dissonance, emotional exhaustion, compassion fatigue, and affective neutrality while also recognising its role in maintaining professional conduct. By adopting this framework, this study offers a more nuanced understanding of how everyday hazards are negotiated through emotional regulation in practice.

## 2 Literature Review

A growing body of research has examined the relationship between occupational hazards and psychological outcomes among nurses. Research across diverse regions like Europe (see Babiarczyk et al. 2020), Asia (Zaman et al. 2023), Australia (Scanlan and Still 2019), Africa (Denge and Rakhudu 2022; Okeafor and Alamina 2018; Shabani et al. 2023), including Ghana (Kaburi et al. 2019; Sarafis et al. 2016) highlights the pervasive nature of psychological hazards among nurses, though their specific forms and consequences vary by context.

The use of quantitative studies, especially cross‐sectional surveys in investigating the psychological hazards encountered by nurses has dominated the extant literature (Khamisa et al. 2015; Molebatsi et al. 2024; Payne et al. 2020; Van der Doef et al. 2012). For instance, Khamisa et al. (2015) found that among nurses in South Africa, stress associated with staff issues was a strong determinant of burnout and job satisfaction. Likewise, Van der Doef et al. (2012) found that burnout scores among East African nurses depicted high levels of depersonalization and emotional exhaustion, with workload being one of the major predictors of burnout and job satisfaction. Like Van der Doef et al. (2012), Molebatsi et al. (2024) recorded the prevalence of depersonalization, emotional exhaustion, and diminished personal accomplishment among nursing staff in Botswana. Notably, they observed a significant role for one's personality in shaping burnout experiences in this context. Moreover, the prevalence of burnout related to clients, work, and personal life, including job satisfaction, among psychiatric nurses in South Africa has been documented (Payne et al. 2020). They established that burnout was negatively associated with job satisfaction.

In the same vein, studies on psychological hazards within the Ghanaian context have been largely quantitative using cross‐sectional surveys (e.g., Lartey et al. 2019; Poku et al. 2020). In their study, they noted a significant negative association between surface acting and job satisfaction among nurses and midwives in Ghana (Lartey et al. 2019). Further, Poku et al. (2020), observed the prevalence of moderate to high levels of emotional exhaustion among registered nurses in Ghana. As well, nurses' practice environment was noted to be a major predictor of emotional exhaustion among this population. Aside from these cross‐sectional surveys, so far, the only qualitative study on emotional labour among nurses in Ghana was the one conducted by Lartey et al. (2020). Using semi‐structured interviews, Lartey et al. (2020) suggested that the nature and scope of the work of nurses and midwives in Ghana demands that they display an ability to manage and regulate their emotions at the work place as in concordance with organizationally prescribed emotions. Evidentially, they provided support for the idea that professional healthcare is linked to emotional experiences encapsulating emotional exhaustion, bullying, abuse, and sadness.

To the best of our knowledge, the evidence on studies on the psychological hazards experienced by nurses within Africa and Ghana are predominantly quantitative with its attendant limitations in providing nuanced understanding of what nurses' experience in the course of duty. Besides, the only qualitative study (i.e., Lartey et al. 2020) is skewed toward only emotional labour and not the broad domain of psychological hazards. Since the study of psychological hazards among nurses within Ghana is at its budding stage, the exploration of this phenomenon using different approaches or designs is imperative. Although the use of semi‐structured interviews in Lartey et al. (2020) affords us the opportunity to hear the voices of these professionals, a way of ascertaining the verity of their responses or experience is to use a design that can provide some form of triangulation to that end. In the current study, the researchers sought to use a focused ethnography that could offer a more suitable way of exploring cultural complexities regarding psychological hazards among nurses in Ghana.

Further, beyond structural and organisational pressures, cultural interpretations of illness and death also shape nurses' psychological experiences (e.g., Brookman‐Amissah 1986; Van der Geest 2004). For example, in Ghanaian settings, deaths are socially appraised as either “good” or “bad” depending on their circumstances, with deaths from old age or common illness seen as acceptable, while those from stigmatised diseases or at a young age are regarded as tragic and problematic (Brookman‐Amissah 1986; Van der Geest 2004). Such cultural evaluations influence how grief is expressed in communities and may affect the emotional burden nurses carry when caring for dying patients. Nurses' exposure to frequent patient deaths, particularly those appraised as “bad,” plausibly intensifies psychological hazards by amplifying sadness, grief, and stress in already demanding work environments (Van der Geest 2004). These findings suggest that occupational hazards are not only physical or environmental risks but also significant psychological stressors that shape nurses' emotional well‐being and professional functioning.

Despite extensive research on occupational stress and emotional labour among nurses, existing studies have largely focused on documenting the prevalence of stress, burnout, and exposure to hazards. However, there is limited qualitative research that examines how everyday occupational hazards translate into psychological experiences and, in turn, shape nurses' behaviour toward patients. Moreover, the application of emotional labour theory in healthcare has often remained narrow, with insufficient attention to broader dimensions such as emotional exhaustion, compassion fatigue, affective neutrality and emotional regulation or coping strategies.

This study addresses this gap by providing an in‐depth qualitative analysis of how occupational hazards generate psychological outcomes that are mediated through multiple dimensions of emotional labour, thereby linking workplace conditions, emotional processes, and nurse–patient interactions in a resource‐constrained setting.

## 3 Methods

### 3.1 Study Design

This study used a focused ethnography. Rather than studying a community's entire culture, as in traditional anthropology, it focuses on a particular aspect of an institution, in this case, a hospital. This method was appropriate for exploring the cultural intricacies of hospital life, enabling prolonged and immersive engagement with participants to develop a richer understanding of their practices and experiences (O'Reilly 2012).

### 3.2 Population and Sample

Since it is impossible to investigate the behaviour of all nurse categories, the study was purposively limited to general nurses working in the male and female medical wards. General nurses were selected because, according to the Ghana Health Service Report (2018), more people report and are admitted to hospitals with general medical conditions such as diabetes, typhoid, malaria, and hypertension, which do not always require specialised nursing services. All participants were registered and had worked for at least a year. After repeated reviews of transcripts and field notes, the first author noted that subsequent data largely confirmed patterns already identified, without yielding new categories, thereby reaching saturation. After the data had saturated, the sample included 31 general nurses and one key informant, the matron.

### 3.3 Instrument and Procedure for Data Collection

A semi‐structured interview guide was used for data collection. The research questions were designed to explore how occupational hazards generate psychological experiences and how these are managed in practice through emotional labour. Some of the questions included: What are the distresses of being a nurse? How do you feel when patients die? Please tell me if you have ever had any work‐related injuries. Please describe the stress related to your job. For participant observation, Spradley's (2016) three‐phase observation approach was adopted. The first, descriptive observation, focuses on everything without sorting the trivial from the important. The second, focused observation, emphasised ongoing activities, such as the ward's daily routines, who does what, and how nurses interact with coworkers and other people. Third, selective observation, focused on what was most important for the study's objectives, such as nurses' responses to patients' calls and needs after experiencing hazards, as well as their verbal and nonverbal interactions with patients and family members.

The first author visited the hospital three times a week, staying through the entire shift in the morning, afternoon, or night. An hour before the end of their shifts, when the hospital was less crowded or had more staff (usually when student nurses had come for clinicals), nurses were interviewed face‐to‐face. The interviews began by gathering participants' sociodemographic information. To gain deeper insight, probes were employed, paying close attention to their body language and facial expressions.

### 3.4 Ethics

Ethical clearance was obtained from the Institutional Review Board (IRB) of a public university (protocol number: ECH 019/20‐21). Upon approval, another consent form was sought from the hospital, which was also approved by the medical superintendent. Subsequently, the hospital matron also approved the presence of the first author in the wards and introduced her to the ward in‐charge (ICs). Informed consent, anonymity, and confidentiality were ensured throughout the study. For example, participants were informed about the goal of the research prior to the interviews, with a focus on the fact that it was conducted only for academic purposes. Some participants provided written consent, while others opted for verbal consent due to concerns about formality. The use of verbal consent was approved by the ethics review board. During the interviews, individuals were informed that participation in the study was entirely optional and that they could withdraw their consent at any time. Each participant was given a pseudonym to protect their identity, and any specific information identifying them was excluded.

### 3.5 Data Analysis

The data obtained were translated, transcribed, analysed, and interpreted using a thematic network analysis approach. The recorded interviews (key informant interviews, in‐depth interviews, and focussed group discussions) were transcribed and translated into English before data analysis. The coding process used in this study was twofold. The first stage was to put the data into themes, that is, global, organising, and basic themes (Attride‐Stirling 2001). For instance, basic themes like death, sickness, and awful sights formed an organising theme: default hazards.

The second stage involved adding quotations for the codes, which NVivo 12 Plus facilitated. For the analysis of the observations, data from the field notes were transcribed on a computer. They were sectioned into themes with the following headings: descriptive observation, focussed observation, and selective observation. For example, descriptive observations included components such as nurses' reporting times, deaths, admissions, and discharges. The best observation on, for example, patient deaths was selected to tell a particular story on that theme.

During the inductive analysis of identifying themes from the data, key concepts such as surface acting, deep acting, and emotional regulation were used as sensitising tools to interpret emerging themes, linking nurses' psychological experiences to their professional behaviour.

### 3.6 Rigour

To ensure rigour, the researcher spent an extended period of 11 months at the hospital. This extended presence, therefore, strengthened the credibility of the data. Credibility and dependability were further enhanced through triangulation, drawing on multiple data collection techniques, including in‐depth interviews, participant observation, key informant interviews, and informal dialogues. Using varied methods provided a more comprehensive perspective than relying on a single approach. A pilot test of the interview guide with three nurses from different facilities also refined the tools and supported methodological rigour. To ensure accurate representation of participants' perspectives, member checking was conducted with some ward in‐charges, while peer debriefing further validated interpretations and enhanced overall trustworthiness.

### 3.7 Researcher Reflexivity

As a Ghanaian social scientist without formal training in clinical nursing, I (the first author) entered the field as an outsider, unfamiliar with both the technical aspects and relational practices of hospital care. My presence was introduced to the participants by the Deputy Director of Nursing Services (DDNS) also referred to as matron. During the COVID‐19 pandemic, and following the DDNS's guidance, I wore hospital scrubs as personal protective equipment. While this was necessary for safety, it occasionally blurred the distinction between my role as a researcher and that of a healthcare professional. To minimise this ethical ambiguity, I adopted a respectful observer stance that allowed for critical observation of routines and hazards and deliberately refrained from involvement in sensitive clinical procedures.

On a few occasions, patients mistook me for a nurse; in such cases, I clarified my role as a researcher. To build rapport and foster trust, I participated in non‐clinical activities such as bed‐making and attended staff workshops. Although some nurses initially responded with caution, my sustained presence over 11 months facilitated openness and candid engagement during interviews and informal conversations.

### 3.8 Profile of Participants

Thirty‐two (32) participants, comprising 27 females and five males, were included in the study. The average age of the participants was 32.6 years, with most being married (*n* = 22). There were four enrolled nurses, nine senior enrolled nurses, five Staff Nurses, five Senior Staff Nurses, two Nursing Officers, five Senior Nursing Officers, one Principal Nursing Officer, and one matron. Excluding the matron, participants had work experience ranging from one to 21 years, with an average length of service of 5 years.

## 4 Findings

Researchers distinguish psychological hazards into hazards that one is likely to encounter (default) and others that one is unlikely to encounter (escapable) and their effect on nurse–patient relationships.

### 4.1 Default Hazards

The authors term ‘default hazards’ as risks arising from an inevitable aspect of the task or job expectations. Nurses are automatically exposed to emotionally taxing situations in some aspects of their work. According to the findings, distress resulting from these hazards sparked feelings of fear, irritation, affection, and exhaustion. These hazards are mainly caused by the fact that nurses must constantly deal with *patient deaths, the ubiquity of sick people, and unpleasant sights*. These hazards stem from the cognitive assessment that such instances or events are unpleasant and distressing, stimulating positive and negative attitudes and behaviours.

#### 4.1.1 Deaths of Patients

During the 11 months of fieldwork, the first author witnessed the deaths of six patients (three men and three women). One of the women, Maimuna, had multiple health issues, including chronic kidney disease and severe hypertension, and had been hospitalised for 2 weeks prior to the author's arrival. Maimuna endured painful procedures for her nasogastric tube feeding, often expressing agony through sounds rather than words. Her 15‐year‐old daughter, who had to leave school to care for her mother, was constantly by her side, assisting with daily tasks. The girl's father limited family visits and placed the full burden of care on her. After 3 months in the ward, Maimuna passed away during a night shift. The first author reflected on the daughter's fruitless struggles and the nursing care provided, particularly during challenging night shifts when they changed Maimuna's diaper. The following morning, the ward IC was seen pacing anxiously, wiping her tears. At the same time, another nurse lay in the nurses' room, also in tears, and the other two nurses appeared sombre as they continued attending to the patients.

This is a common psychological hazard for most medical professionals, particularly nurses. Participants reported feeling powerless during the dying process because they were unsure of what to do after all attempts to save these patients had failed. One said:

There have been times, even a week after death, when I keep brooding over the events. Mentally, I see the dead patient when I come to work, and I will be thinking about whether I faulted in any way that could have led to the patient passing so imagine if it was really my fault! The trauma would be unbearable! (Awurama, female, Staff Nurse).

This account shows how unresolved grief and guilt undermine surface acting, highlighting the psychological toll of deep emotional investment. Participants also stated that a nurse's feelings of grief over a patient's death could lead to inappropriate behaviour when further provoked. Bernice demonstrates:

One patient died unexpectedly, and the way it all happened affected me mentally. Then, another patient's relative came in for the third time to ask me a question I had already answered earlier. I shouted, ‘Be patient, madam!’ She made me mad, and I reacted in that way because of the death earlier. Not only can it affect your output, but you can also act unprofessionally (Bernice, female, Senior Staff Nurse).

The accounts highlight that the death of a patient can significantly influence nurses' behaviour. Although many nurses have encountered numerous deaths in their careers, some struggle to cope with and show signs of distress with each new loss. This reaction often stems from the personal connections they form with patients and the emotional weight these relationships carry. Factors contributing to this distress include familiarity with the patient, similarity of the condition to that of the nurse's experience, the patient's manners, and the traumatic nature of the death.

The first characteristic was common among individuals who spent a significant amount of time in the ward. As in Maimuna's case, patients who had been admitted for an extended period of time, say, a month or more, were regarded as ‘members of the house.’ This was also applicable to patients who were frequently admitted and discharged.

Another notable characteristic is well‐mannered or courteous patients. The participants noted that these patients were humble and cooperative, making care easier for nurses. Just as nurses dislike impolite patients, they naturally develop affection for well‐behaved patients. Consequently, the loss of cherished patients is sorrowful.

I lost a 72‐year‐old patient. He was hilarious and always wanted to chat. He would sometimes walk to you, saying, “Aunty nurse, I want to chat with you.” He became my friend and would not allow anyone to check his vitals if I were on duty. That day, he died, I really cried as if I had lost my father. Some people make us feel like we are their family (Kate, female, Enrolled Nurse).

This narrative exemplifies the profound emotions inherent in emotional labour theory, illustrating how genuine bonds can heighten vulnerability to grief and emotional exhaustion.

Male nurses formed similar connections and grieved the loss of certain patients; however, their expressions of grief differed from those of female nurses. Men tended to discuss their feelings, while women not only discussed them but also showed signs of moodiness and sorrow.

As a coping mechanism, only a few nurses acknowledged the need for psychologists to help them with their grief. Many felt that such emotions were inherent to their job, while others were reluctant to seek help for fear of being labelled as depressed. Many participants acknowledged that the pain and heartbreak associated with their profession could be overwhelming for some nurses. Yet, they also felt the need to conceal their grief or surface acting to maintain a professional demeanour, as the theory posits. One explained:

IC has been very worried about the man on bed 8, whose leg needs to be amputated, but she speaks to the relatives without emotion, so they feel we do inhumane things, but trust me, it's very depressing. One thing I keep saying is nurses need psychologists. We need counselling! We are one of the most depressed professionals you could ever find…I tell you! (Georgina, female, Senior Enrolled Nurse).

Georgina's account illustrates surface acting, where concealing distress maintains professionalism but risks being misinterpreted as inhumanity by families. Nevertheless, some nurses felt no emotional response to the deaths of patients, particularly those who did not have the aforementioned characteristics. Participants admitted that many nurses developed a form of sensory adaptation, becoming somewhat indifferent to these frequent events, such that death no longer affected them. For instance, one said:

I do not know if it's because we have practiced for a long time. Some of us are emotionless when we see the dead, and I feel we need periodic vacations to refresh. It is not like we do not have emotions, but we feel less of them than others. We are hardened, so death is no longer news (Davida, female, Senior Staff Nurse).

Davida's narrative illustrates emotional numbing as protective detachment within emotional labour, and also highlighting burnout risk.

Deaths triggered grief, guilt, and feelings of powerlessness. Nurses were expected to maintain composure, often relying on surface acting to suppress sorrow when speaking to relatives. Others engaged in deep acting, inducing empathy to sustain compassion even when overwhelmed. Emotional regulation included concealing emotions, seeking peer support, and spiritually reinterpreting death as inevitable. Some nurses also developed sensory adaptation or emotional detachment (affective neutrality) over time as a form of protective coping, although this sometimes risked seeming unempathetic.

#### 4.1.2 The Ubiquity of Sick People

The second default hazard arose from the ubiquity of the sick. The first author frequently felt disheartened by the sight of sick people, an unfamiliar experience. By contrast, many nurses found this to be a normal part of their lives. Most participants noted that newer, less experienced nurses were often more emotionally affected by patients' situations than more experienced ones. However, the findings indicated that emotional responses were influenced not only by experience but also by factors such as the severity of a patient's illness, their age, and even their resemblance to familiar individuals.

This finding on the ubiquity of sick people, another default hazard, evokes feelings of distress, sympathy, and connection, particularly in patients with certain traits. While participants claimed to treat all patients fairly, they sometimes felt a stronger emotional attachment to specific individuals, resulting in a more compassionate demeanour. Georgina, who associates youth with good health, acknowledges the gravity of patients' condition.

It's depressing…it's depressing! Sometimes, when you see a 30‐year‐old with diabetes, a chronic one! It's sad. We are humans ooo. People think we have no empathy, but we do not show it because we cannot attend to the next patient if we do (female, Senior Enrolled Nurse).

This demonstrates suppressed empathy, characterised as surface acting, which helps continue care but is costly in terms of emotional dissonance and the risk of burnout or emotional exhaustion.

According to the participants, the age of the patients tended to induce the emotions and distress experienced by healthcare providers. Patients aged 13 years and older are admitted to medical wards, and participants admitted that they often feel a deeper emotional connection to both younger and elderly patients, viewing them as particularly vulnerable individuals who require more attention and empathy. This sense of connection fosters positive attitudes toward these patients. Diana (female, Nursing Officer) expressed her attachment to elderly patients: “I get attached to the elderly because of my great‐grandmother. I feel they are very helpless to the core.”

A 13‐year‐old boy presented with diabetes mellitus and was often admitted. His family did not have much, so I sometimes bought insulin. Even when I am absent, he would call, and I would buy it for him. I feel emotional whenever I see him (Betty, Male ward IC).

Betty's story exemplifies deep acting, where genuine empathy drives personal sacrifice, but also risks overinvestment and blurred boundaries.

Many participants highlighted that a nurse's personal history can shape their behaviour toward patients. For instance, some nurses recalled similar experiences in their own lives or among friends and family members, influencing how they related to patients facing similar conditions. Francis recounted his attachment to an alcoholic patient who reminded him of his father, expressing a strong desire for the patient to recover despite the damage caused by alcohol. Francis recalled:

He was a known alcoholic, and he was like a father figure to me because he was an age mate with my father… that's all I saw in him, my father. I got so attached to him. I wanted him to survive to quit it, but the alcohol had destroyed his kidneys and liver. When I see such patients, I feel emotional (Male, Senior Enrolled Nurse).

The nurse's paternal identification with the patient illustrates deep acting, demonstrating how personal associations can intensify compassion but also blur professional boundaries.

Nurses' emotional involvement can lead to overwhelming responsibilities, particularly for patients with whom they have a close relationship. Such attachments may hinder these patients' ability to manage crises and affect their overall performance. In addition, while nurses may spend more time with these patients, others may feel neglected.

Some participants acknowledged their emotional ties with certain patients but chose not to display these feelings, fearing that patients or their families might exploit them.

We get attached to them all the time, but we do not show it because immediately a relative realises that you are ‘attached’ to their relation (the patient), they ask for too many favours. They want to take your phone number; they want you to favour them in the bills; they even want you to come home, so we do not show it (Edna, female, Staff Nurse).

Edna's account demonstrates the strategic use of surface acting to protect personal boundaries, highlighting how emotional labour is shaped not only by professionalism but also by social pressures from patients' families. It suggests that some nurses deliberately show indifference to patients and their relatives in order to avoid any future commitments.

In sum, constant exposure to illness evoked distress and compassion, especially when patients resembled family members or were young or elderly. Nurses balanced empathy with emotional neutrality, using surface acting to prevent over‐attachment that could impair their professional performance. Emotional regulation included acts of kindness (e.g., buying medication), while others intentionally showed indifference to avoid exploitation by families. These behaviours demonstrate emotional labour as both a tool for compassion and self‐protection.

#### 4.1.3 Unpleasant Sights

Participants found certain ‘unpleasant sights’ distressing, contributing to negative attitudes in their work. While the ubiquity of sick people is about the constant presence and visibility of patients with varying illnesses in the ward, unpleasant sights denote specific sensory encounters with bodily fluids, wounds, infections, and other graphic conditions that provoke disgust or discomfort and demand immediate emotional regulation. Again, while both are psychological hazards, the former arises from continuous exposure to human suffering, and the latter from acute encounters with disturbing physical conditions.

The first author described experiencing cultural shock on the first day at the hospital while observing a wound dressing in a male ward. The patient, an elderly man with diabetes, had an amputated leg with a severely infected stump wrapped in a bandage. As the nurse began to unwrap the bandages, the smell became overwhelming and the patient moaned in pain, “oopla mi eh” … “oopla mi eh” (you are hurting me…you are hurting me). The sight of worms emerging from the wound was shocking, and the author struggled to maintain composure while observing the dressing. Many participants admitted that the presence of worms in wounds was particularly distressing, along with other unpleasant sights such as vomiting, blood, pus, and soiled diapers. Augustina complained:

I remember the day I was asked to change a woman's diaper, my dear, how does even a baby's loaded diaper look, let alone an adult's? What I saw was very inconvenient, but I had to look at the colour, smell, and chart it. Sometimes, you must pour it into a measuring cup to measure it too! That day, it was not easy for me [Both laughs] (female, Staff Nurse).

Her narrative reveals how humour serves as a coping strategy, masking disgust and highlighting the role of surface acting in managing unpleasant sights. Mabel (female, Enrolled Nurse) also expressed: “I saw one woman who had sores from her waist to her legs. It was terrible! One colleague saw her and collapsed. If it's your first time, you can collapse, vomit or cry.”

Sometimes when I get to work and have not eaten, I cannot eat during the entire shift because of the encounter with suffering patients, dead bodies, blood, vomit, and sores! One time, I cried while consoling my patient. Psychologically, it gets to a time that you become emotional about many things. When you get to work, you are already down for the day (Joana, female, Nursing Officer).

Joana's account demonstrates cumulative exhaustion and blurred boundaries, underscoring the need for systemic relief measures, such as debriefing and rest. While nurses often develop a certain tolerance for the sights of death and illness, most participants acknowledged moments of civil inattentiveness toward the unpleasant sights they encountered. Exposure to wounds, blood, and human waste produced strong feelings of disgust and fatigue. Nurses often hid visible emotions (surface acting) while providing care. Emotional regulation included using humour with colleagues, temporarily withdrawing from eating, and relying on peer encouragement. Over time, some developed tolerance, although others reported burnout. This aligns with Hochschild's assertion that prolonged surface acting leads to emotional exhaustion.

### 4.2 Escapable Hazards

The authors define ‘escapable hazards’ as work‐related risks one may never encounter. Although many participants had experienced some of them, some had not, due to proper care. These had to do with work‐related injury and shift‐work maladaptation syndrome, characterised by anxiety, fear, and anger, which trigger undesirable behaviours toward patients in some instances.

#### 4.2.1 Work‐Related Injury

Work‐related injuries commonly encountered by nurses include falls, cuts from sharp objects, and musculoskeletal disorders from lifting patients or heavy loads. Needle pricks were the most frequent injury in the hospital, with many participants admitting to experiencing at least one needle prick in the past year. The study found that junior nurses were more likely to suffer from needle‐prick injuries than their senior counterparts, primarily due to their lack of experience.

Patients who had needle pricks were required to take antibiotics and undergo various tests to prevent complications. During this period, they often experience mental distress, which could adversely impact their job performance and attitudes toward patients. Some FGD participants shared their personal experiences with these challenges:

I was discarding the needle I used when it pricked me. What even hurt me most was my inability to identify whose needle it was, so I had to take retro medications. Being pregnant at the time, taking the drugs was not easy. For about a week, my blood pressure was always high. Thankfully, all the tests I did every two weeks were negative, but I was still in fear until the sixth month, which confirmed that I was genuinely negative (Martha, female, Senior Enrolled Nurse).

Martha's case shows how needle pricks during pregnancy intensify fear and anxiety, highlighting the limits of coping. Diana also recounts the story of a colleague and how this could affect relations with patients:

A colleague had a needle prick, and the patient was a retro. She said she told the patient to be still, and just after injecting him, he moved, and it pricked her. She became so angry and abhorred the patient. You should have seen what she did to the patient! She really insulted and dealt with him, and I do not blame her because she could have had HIV. Subsequently, when you tell her to give an injection, she refuses. I run the ward with her on the same shifts a lot of time, and anything to do with injectables; she is not ready to do (female, Nursing Officer).

These quotations indicate that nurses often experience anxiety about needle pricks, primarily due to the fear of contracting HIV/AIDS and other blood‐borne pathogens that pose health risks. This anxiety can negatively impact the attitudes and behaviours of affected nurses toward patients involved in such incidents, leading them to ignore these patients in future situations, as seen above.

Another type of injury that some nurses report is a musculoskeletal injury resulting from lifting and falling. Nurses must frequently adjust their body positions to move away from their normal anatomical postures in order to perform their tasks effectively. In certain procedures, they may need to maintain these deviant positions for extended periods, increasing the likelihood of developing musculoskeletal disorders. The participants expressed concerns about the daily consequences of lifting heavy loads, especially when they were required to lift patients without the assistance of orderlies or family members.

One day we were lifting a patient when I felt a lock in my waist. Now, I have waist and back pain, which has brought many difficulties. Sometimes the pain radiates to my neck and head…. (Mabel, female, Enrolled Nurse).

This highlights how musculoskeletal injuries often exceed individual coping strategies, underscoring the need for ergonomic support and systemic interventions. Sandra, an assistant IC, recalled the following predicament:

A very heavy patient tried to get out of bed and fell. I was the only one around and could not ask the remaining patients to assist me in lifting her. I fetched another nurse from the next ward, but we were both a few months postpartum. As we struggled to carry her onto the bed, I felt a crack and sharp pain in my back, and until now, I have had severe back pain. I do not attempt to lift a patient, not because I am unwilling to do it; I simply cannot do it (Female, Senior Nursing Officer).

This highlights a significant deficiency in hospital wards regarding patient safety, particularly the issue of patients falling out of bed. Although there were electric beds with adjustable rails for non‐ambulant patients, they were inadequate. In cases where beds lack rails, the patients' limbs are sometimes tied down with cloth to prevent falls. However, such incidents are still seen as failures and barriers to quality healthcare, and they pose risks to nurses' health, consequently affecting the quality of healthcare.

I am aware that the Ghana Health Service has implemented occupational health and safety measures, which we are required to comply with; however, no one regards them as a priority since there are no alternative options for patients to be lifted. Due to some of these issues, I have lost my passion for nursing, which has affected my commitment (Stella, Female, Staff Nurse).

The account illustrates how inadequate institutional safety measures erode passion and commitment, making emotional labour unsustainable. The matron indicated that lifting is now primarily the responsibility of orderlies, especially because these physical demands have affected many female nurses.

Participants also shared their experiences of injuries from falls, particularly when responding to emergencies due to faulty intercoms or slips caused by spills. One nurse experienced hip dislocation from such a fall, resulting in chronic pain. Additionally, some nurses faced the discomfort of being unintentionally vomited on by patients, which, while not injurious, raised concerns about potential infections. Nurses who experienced falls, waist pain, needle pricks, and other injuries often refused or ignored duties that could result in further injuries because they found it impossible to suppress such emotions. This affected their commitment to their jobs, especially given the structural challenges and limited compensation, which included only a 2‐week period off duty. The hospital matron had this to say about her nurses' injuries in the ward.

… My headache is the needle pricks. If you are careful, you will not be pricked. The falls are better now, because the odleys are always around to clean, but my issue is the pricks (female, DDNS).

This view highlights leadership recognition of persistent needle prick risks. Three workshops were held during the research, but the matron was particularly interested in the injection safety workshop, indicating her awareness of frequent reports of needle pricks among her staff. She enthusiastically drove nurses from the wards to the conference room because she constantly received reports of pricks. This observation became much more meaningful following the interviews.

Needle pricks triggered fear of HIV/AIDS, causing anger and anxiety that were hard to control. Emotional effort was strained, with some nurses retaliating against patients or refusing injection duties afterward. Emotional regulation included avoiding risky tasks, seeking medical reassurance through tests, and sharing experiences in workshops. Musculoskeletal injuries also lowered morale, with coping efforts involving delegating lifting tasks to orderlies, peer assistance, and advocating for better equipment.

#### 4.2.2 Shift‐Work Maladaptation Syndrome

The nursing staff is organised into three shifts to ensure patients receive comprehensive healthcare around the clock. The first group of nurses began their shift at 8 am and concluded at 2 pm, providing care during the day. Following them, a second group took over in the afternoon, starting at 2 pm and ending at 8 pm, ensuring continuity of care in the evening. Finally, a third group worked the night shift from 8 pm to 8 am, covering the overnight hours when patient needs may arise.

Most nurses worked both day and night shifts, depending on the roster, while some nurses chose to work only day or night shifts due to other commitments, such as schooling, working at another health facility (locums), or pregnancy. The participants expressed significant concerns about the impact of night shifts, particularly on their overall well‐being. Many nurses described night shifts as especially stressful and disruptive, making it difficult to adjust their sleep patterns and manage their fatigue. This disruption not only hampers their ability to provide optimal patient care but also negatively influences their capacity to maintain positive interactions and behaviours both at work and in their personal lives. They reported that working during these hours not only adversely affected their physical health but also their safety and intimate relationships with their partners.

For example, Augustina shared, “There was a time I had to take sleeping pills to get rest at home. I just can't sleep.” Similarly, Jessica, a participant in the FGD, expressed her frustration regarding the afternoon shift.

My colleague reaches home at approximately 11 p.m. after every afternoon shift. The following day, you expect the person to work a morning shift at 8 am. When they come, they are visibly tired, which affects delivery. In nursing, our headaches are the shifts, the roster, and the timing! Every day, nurses cry about the roster. The stress is just too much (Jessica, Senior Enrolled Nurse).

Jessica's case illustrates how poor rostering exacerbates fatigue and undermines coping mechanisms, reinforcing the need for institutional roster reforms. Martha, another participant of the FGD, also narrated:

Last week, there was a student in the female ward whose bag was snatched on the roadside. Apparently, the nurse for the night shift did not report early, and the nurse on duty, who could have relieved the student to go, did not release her until about 9:30 pm instead of 8 pm when the nurse for the night shift arrived. According to the student, as she stood awaiting a taxi, she was attacked by two men with a knife, and all her belongings were taken, including her bag and phone. Sometimes we are at risk just by being nurses because the shifts and timing don't help us (Female, Senior Enrolled Nurse).

These findings contribute to understanding of shiftwork maladaptation syndrome and its effects on sleep and safety. For instance, if a nurse finishes an afternoon shift at 8 pm and must walk to a bus stop at night to use public transport, they may be vulnerable to attacks by criminals, as suggested by the previous quote. Therefore, if their work jeopardises their safety, the quality of healthcare they provide may be adversely affected. Nurses who usually worked day shifts (such as ICs and nursing mothers) were more likely to escape the aforementioned hazards than those who opted for only night shifts (due to school or locums). This disparity highlights the significant toll night shifts can make on nursing staff.

Night shifts led to fatigue, sleep disruptions, and safety issues. Nurses reported using surface acting to seem attentive while feeling internally exhausted. Coping strategies included using sleep aids, such as pills, using humour with colleagues, informal shift swaps, and seeking more flexible schedules. Some also emphasised the importance of peer support in handling the psychological stress. Despite these efforts, emotional dissonance persisted, leading to emotional exhaustion and strained relationships both at work and at home.

## 5 Discussion

This study aimed to investigate how occupational hazards experienced by nurses influence psychological outcomes that impact nurse–patient relationships. Research examining the distressing experiences of nurses regarding patient deaths, particularly during their first encounters, revealed similar emotions, such as shock, grief, fear, denial, guilt, helplessness, and a loss of confidence (Kostka et al. 2021). While these studies focused on the distress experienced by early career nurses during their first encounters with patient deaths, this study highlights that experienced nurses also face similar emotional challenges from repeated exposure to such events. The research supports the idea that women tend to exhibit greater emotional empathy, likely due to socialisation that encourages expressiveness, unlike men, who are often taught to control their emotions (Cunico et al.'s 2012). These distressing situations can adversely affect nurses' emotional and mental well‐being (Zaman et al. 2023), leading to negative attitudes and poor behaviour toward patients and their families. Moreover, within the Ghanaian society, an appraisal of a person's death as either “good” or “bad” could affect their emotional response, irrespective of the age or experience of the individual (Brookman‐Amissah 1986; Van der Geest 2004). Nurses' appraisal of the illness of their patients as terrible, leading to their death, is likely to evoke negative emotions of emotional distress, irrespective of the rank of the nurse.

It has been noted that concealing grief is seen as a mark of professionalism, as nurses are expected to present a calm and competent demeanour (Kirk et al. 2021). This surface acting could be emotionally exhausting, and the emotional disconnection can lead nurses to perceive themselves as uncaring. In such cases, nurses had to induce feelings (deep acting) of empathy to appear empathetic, as Hochschild (2003) posited. Therefore, this study suggests that while some nurses manage their emotions to maintain professionalism, others may not feel any emotions, reinforcing the notion that nursing is an emotionally challenging profession.

Also, the ubiquity of sickness was identified as a default hazard. Mottaghi et al. (2019) noted that nurses frequently encounter situations that require empathy, which can lead to overwhelming feelings of responsibility toward patients. This emotional connection may hinder nurses' ability to effectively manage crises for certain patients, potentially affecting their performance. As nurses devote a significant amount of time to these patients, others may feel neglected. Consequently, maintaining affective neutrality in the nurse‐client relationship is advised (Parsons 2014). Regarding unpleasant sights, as Atinyagrika Adugbire and Aziato (2018) reported, some nurses may display unprofessional behaviours, such as refusing to treat infected wounds or caring for patients with unpleasant sores due to the discomfort and emotional exhaustion they experience. This raises concerns about occupational burnout, in which prolonged exposure to emotional burdens results in compassion fatigue and significant emotional, mental, and physical exhaustion (Norouzi et al. 2022) as well as employee safety concerns (Hong et al. 2024).

Further, certain hazards that could be avoided (escapable), with many being work‐related, were identified. Common hazards included injections, musculoskeletal disorders, and injuries (Amare et al. 2021; Misiak et al. 2020). Although these represent potential risks for nurses, not all participants reported encountering them. The overall worldwide prevalence of needlestick injuries among nurses is 40.97%. The WHO regions had the highest prevalence in Southeast Asia (49.9%) and the lowest in the USA (25.1%). The pooled prevalence in developed and developing countries is 30.5% and 46.6%, respectively (Abdelmalik et al. 2023).

This trend is also evident in Ghana and various international clinical settings. Additionally, these studies indicated that needle‐stick injuries and exposure to infectious bodily fluids are common among auxiliary nurses, while professional nurses mainly face assaults, falls, and equipment‐related injuries. However, this distinction was not observed in this study. When nurses retaliate against patients who cause them the pricks, they diverge from the expected standards of care. According to the participants, suppressing or managing feelings (surface acting) in these situations is nearly impossible, indicating stress in emotional labour (Hochschild 1983).

Moreover, nurses often spend long hours on their feet and may need to lift patients who weigh significantly more than they can safely manage. Additionally, in certain procedures, awkward postures must be maintained for extended periods, which heightens the risk of work‐related musculoskeletal disorders [WMSD] (Rathore et al. 2017). These disorders represent a significant public health issue among healthcare workers and are increasingly being recognised as a common cause of morbidity, especially among nurses (Kgakge et al. 2021). This systemic oversight impacts their willingness to provide similar assistance to patients, later finding the suppression of such emotions almost impossible, as Hochschild (1983) posits.

Again, shift‐work maladaptation syndrome has been identified as a potentially escapable hazard that can result in sleep deprivation, fatigue, and disrupted biological cycles, impairing their ability to work, which is consistent with current research in Bangladesh, China, and other countries (Fink 2024; Wu et al. 2024). Shift work is not exclusive to Ghana; however, the specific structural and individual challenges nurses face make it particularly difficult, as highlighted in the current study. While the existing literature highlights the health consequences of shift work, this study underscores a critical safety concern. The risk of criminal attacks adds to the stress and hazards of shift work in Ghana, ultimately affecting the quality of healthcare delivery. In such cases, nurses must constantly evoke feelings of care when they encounter these challenges in their profession (Hochschild 1983).

The study extends emotional labour theory beyond surface and deep acting by showing that nurses also experience emotional exhaustion, compassion fatigue, affective neutrality, and ongoing emotional regulation. These broader dimensions highlight how nurses cope with sustained emotional demands, thereby offering a more comprehensive application of emotional labour theory in healthcare settings.

## 6 Conclusion

This study has shown that occupational hazards in hospital wards, whether default or escapable, carry profound psychological consequences for nurses that directly shape their interactions with patients and families. By applying Hochschild's emotional labour theory, the analysis revealed how nurses manage distressing experiences through surface acting, deep acting, and a range of coping strategies. Deaths, constant exposure to sickness, and unpleasant sights triggered grief, disgust, and compassion, often suppressed to maintain professionalism, while injuries and shift‐work maladaptation heightened fear, fatigue, and frustration. Nurses responded with both emotion‐focused coping (suppression, detachment, spirituality, peer support) and problem‐focused coping (task delegation, safety workshops, roster adjustments). However, these personal strategies often proved unsustainable, creating emotional dissonance and, in many cases, burnout.

The findings extend existing literature by demonstrating that emotional labour is the mediating process linking hazards, coping, and nurse behaviour. Notably, the study highlights that the responsibility for managing these hazards cannot be solely placed on individual nurses. Without systemic interventions, nurses are left to rely on fragile coping mechanisms that erode their well‐being and professional commitment.

Sustaining quality healthcare in resource‐constrained settings requires more than individual resilience. It calls for institutional commitment to recognising emotional labour as integral to nursing practice and to providing comprehensive support systems. Specific strategies include: establishing psychological counselling services and regular debriefing sessions for nurses; ensuring ergonomic equipment and adequate staffing to reduce musculoskeletal injuries; implementing effective injection safety protocols and continuous occupational health training; introducing flexible and fair rostering systems to mitigate shift‐work maladaptation; and enhancing security measures for staff leaving late shifts.

These findings also provide important implications for policy and training. Policymakers can integrate occupational health and emotional labour awareness into national nursing standards and accreditation systems. Nursing curricula should also include structured modules on coping strategies, resilience‐building, and safe work practices. At the system level, health service managers should monitor compliance with occupational health guidelines and allocate resources for continuous professional development. Strengthening such supports will not only safeguard nurses' psychological health but also improve their professional commitment, ultimately enhancing the quality and humanity of patient care.

Although Hochschild's (1983, 2003) emotional labour theory provides practical application in demonstrating how surface and deep acting are mobilised in everyday nursing practice, the study acknowledges critiques that emotional labour theory can underplay structural constraints by focusing on individual strategies. Our findings address this by linking emotional labour to system‐level shortcomings such as poor rostering, inadequate safety equipment, and limited psychosocial support, thereby demonstrating that emotional labour is not only an individual burden but also a reflection of institutional responsibility.

### 6.1 Limitations and Future Research

The study focused on nurses' self‐reported coping strategies and did not include patient or relative perspectives, which could have provided a fuller picture of how psychological hazards influence nurse–patient interactions.

Future research should explore how families perceive nurses' emotional labour and behavioural responses. This would provide a more holistic understanding of how occupational hazards influence nurse–patient interactions.

## Author Contributions

P.N.B.K. collected the ethnographic data. P.N.B.K. and F.P. prepared the manuscript draft. All authors were involved in conceptualising and reviewing the final manuscript, and all authors approved the final version to be published.

## Funding

This work was supported by the BANGA‐Africa Project Thesis Completion Grant.

## Ethics Statement

The Institutional Review Board (IRB) of the University of Ghana approved this study (protocol number: ECH 019/20‐21).

## Conflicts of Interest

The authors declare no conflicts of interest.

## Data Availability

Due to privacy and ethical concerns, neither the data nor the source of the data can be made available.
